# *CASP8* variants D302H and −652 6N ins/del do not influence the risk of colorectal cancer in the United Kingdom population

**DOI:** 10.1038/sj.bjc.6604314

**Published:** 2008-03-25

**Authors:** A M Pittman, P Broderick, K Sullivan, S Fielding, E Webb, S Penegar, I Tomlinson, R S Houlston

**Affiliations:** 1 Section of Cancer Genetics, Institute of Cancer Research, Sutton, UK; 2 Molecular and Population Genetics Laboratory, London Research Institute, Cancer Research UK, London, UK

**Keywords:** *CASP8*, colorectal cancer, risk, polymorphism

## Abstract

Polymorphisms in *CASP8* at 2q33.1 have been associated with the risk of developing cancer, specifically, the D302H variant (rs1045485) with breast cancer in the European population and the −652 6N ins/del promoter variant (rs3834129) with multiple tumours including colorectal cancer (CRC) in the Chinese population. We evaluated the relationship between −652 6N ins/del and D302H variants and risk of developing CRC in the UK population by genotyping 4016 cases and 3749 controls. Both variants showed no evidence of an association with risk of developing CRC (*P*=0.42 and 0.22, respectively). In contrast, the recently identified CRC susceptibility allele rs6983267 mapping to 8q24 was significantly associated with disease risk (*P*=8.94 × 10^−8^). It is thus very unlikely that variation in *CASP8* defined by −652 6N ins/del or D302H influences the risk of CRC in European populations. The implications of our findings both in terms of population-specific effects and publication bias are discussed.

Although inherited susceptibility is responsible for ∼30% of all colorectal cancers (CRCs) (Lichtenstein *et al*, 2000), high-penetrance, germline mutations in APC, the mismatch repair (MMR) genes, MUTYH/MYH, SMAD4, ALK3 and STK11/LKB1 account for <5% of cases (Aaltonen *et al*, 2007). Much of the remaining variation in genetic risk is likely to be explained by combinations of more common, lower-penetrance variants.

Although such common alleles may only confer modest differences in CRC risk through interaction with other common alleles, some individuals will be placed at a significant risk. Hence, screening for a battery of such markers potentially has clinical utility. It is, however, clear that robust validation of novel CRC predisposition loci is required to avoid spurious testing.

The availability of high-resolution linkage disequilibrium (LD) maps, and consequently of sets of tagging SNPs that capture much of the common sequence variation, allows whole-genome-wide association studies (GWAS) for disease associations to be efficiently conducted. This approach is attractive as it is unbiased and does not depend upon prior knowledge of function or presumptive involvement of any gene in disease causation. Furthermore, it minimises the possibility of failing to identify important variants in hitherto unstudied genes. Recent GWAS have vindicated the assertion of the ‘common-disease common-variant’ hypothesis providing evidence for common disease variants for CRC mapping to 8q24 (Haiman *et al*, 2007; Tomlinson *et al*, 2007; Zanke *et al*, 2007) and *SMAD7* (Broderick *et al*, 2007). The applicability of the GWA strategy, however, depends heavily on the robustness of tagging, and for many regions of the genome this may not be optimal. This is a potentially serious limitation for regions of the genome to which strong candidate genes map and are not properly tagged.

Caspase 8 (*CASP8*; MIM 601763) is a key regulator of apoptosis or programmed cell death, an essential defense mechanism against hyperproliferation and malignancy. Polymorphic variation in *CASP8* has been reported to influence cancer risk. The variant D302H (rs1045485) has been associated with risk of breast cancer in the European population (Cox *et al*, 2007). Furthermore, the −652 6N ins/del promoter variant (rs3834129) has been associated with the risk of developing multiple tumour types, including CRC in the Chinese population (Sun *et al*, 2007). It has been asserted that this specific variant has direct functional effects on cancer risk on the basis that the deletion destroys a stimulatory protein 1 binding site and decreases *CASP8* transcription (Sun *et al*, 2007). Moreover, biochemical analyses have shown that T lymphocytes with the deletion variant have a reduced caspase-8 activity and activation-induced cell death upon stimulation with cancer cell antigens (Sun *et al*, 2007).

To confirm or refute the purported association between −652 6N ins/del and CRC risk and evaluate the relationship between D302H and CRC risk, we genotyped 4016 cases and 3749 controls ascertained from the UK population. Risk estimates were compared with those associated with the 8q24 risk allele rs6983267 using previously generated data (Tomlinson *et al*, 2007).

## MATERIALS AND METHODS

### Subjects

Cases (1930 males, 2086 females; mean age at diagnosis 59.5 years; s.d.±8.7) were ascertained through the National Study of Colorectal Cancer Genetics (NSCCG) (Penegar *et al*, 2007). Healthy control individuals (2092 males, 1657 females; mean age 60 years; s.d.±10.8) were recruited from NSCCG (*n*=1843), the Genetic Lung Cancer Predisposition Study (1999–2004; *n*=479) and the Royal Marsden Hospital Trust/Institute of Cancer Research Family History and DNA Registry (1999–2004; *n*=1427) (Tomlinson *et al*, 2007). Cases and controls were British Caucasians, and there were no obvious differences in the demography of cases and controls in terms of place of residence within the UK. The study was conducted with ethics committee approval (MREC/98/2/67; MREC02/0/97) in accordance with the tenets of the Declaration of Helsinki, and written informed consent was obtained from all subjects.

### Genotyping

DNA was extracted from samples using conventional methodologies and quantified using PicoGreen (Invitrogen Corp., Carlsbad, CA, USA). Genotyping of D302H (rs1045485) and −652 6N ins/del (rs3834129) was conducted by competitive allele-specific PCR KASPar chemistry (KBiosciences Ltd, Hertfordshire, UK). For rs1045485, allele-specific primers were: GAAGGTGACCAAGTTCATGCTAGATTTGCTCTACTGTGCAGTCATC and GAAGGTCGGAGTCAACGGATTAGATTTGCTCTACTGTGCAGTCATG; common primer: GACCACGACCTTTGAAGAGCTTCAT. For rs3834129, allele-specific primers were: GAAGGTCGGAGTCAACGGATTGCCATAGYAATTCTTGCTCTGCCAA and GAAGGTGACCAAGTTCATGCTGCCATAGYAATTCTTGCTCTGCCAC; common primer: CACTGAGACGTTAAGTAACTTGCCCAA. Genotyping quality control was tested using duplicate DNA samples within studies and SNP assays, together with direct sequencing of subsets of samples to confirm genotyping accuracy. For both SNPs, 99.6% concordant results were obtained.

### Statistical analyses

Statistical analyses were undertaken using STATA Software (StataCorp LP, College Station, TX, USA). To test for population stratification, the distribution of genotypes in controls was tested for a departure from Hardy–Weinberg equilibrium (HWE). Logistic regression was used to calculate odds ratios (ORs) and the associated 95% confidence intervals (CIs). Power calculations were undertaken on the basis of proportions. In all analyses, *P*-value of 0.05 was considered significant. Linkage disequilibrium between genotypes was enumerated using the metric *R*^2^. This was estimated using Haploview software.

## RESULTS AND DISCUSSION

D302H (rs1045485) and −652 6N ins/del (rs3834129) genotypes were obtained for 96% (3843) and 97% (3879) of CRC cases and 97% (3631) and 98% (3661) of controls, respectively; hence, there was no evidence of any systematic bias in genotyping. Furthermore, there was no evidence of population stratification, as the genotype distribution in controls for both variants satisfied HWE (Table 1). The frequency of the 302H allele (0.132) in our study was similar to that reported in a other European populations (0.130 (Cox *et al*, 2007) and 0.125 CEPH (http://www.ncbi.nlm.nih.gov/projects/SNP/)). In the Chinese population, this SNP has, however, been documented to be nomomorphic. The frequency of −652 6N del allele in controls was 0.501, again similar to previously published data on Europeans. It is, however, markedly different to that observed in the Chinese population in which the frequency is ∼25%.

The frequencies of both variants were not significantly different in cases and controls (allele test of significance, *P*=0.423 and 0.216 respectively; Table 1). There was not strong LD between rs3834129 and rs1045485 (*r*^2^=0.19). By comparison, the 8q24 variant rs6983267 displayed a strong association with CRC risk (*P*=8.94 × 10^−8^).

Based on the number of cases and controls analyzed and the population frequencies of the rare alleles of each polymorphism, our study has ∼90% power to demonstrate an OR of 1.2 associated with each variant stipulating a *P*-value of 0.05. Hence, while the D302H polymorphism has been robustly shown to influence breast cancer risk (Frank *et al*, 2006), our study indicates that the variant is unlikely to affect risk of CRC and hence its effect on cancer risks is not generic. Nevertheless, we cannot exclude the possibility that each variant is associated with more modest risks of CRC (i.e. <1.2, based upon the upper 95% CI for ORs).

The −652 6N ins/del variant has been reported to influence both breast and CRC in the Chinese population. A recent study of breast cancer with the Europeans has failed to confirm an association with breast cancer risk (Frank *et al*, 2007).

In our study, genotyping was conducted by allele-specific PCR, generally acknowledged to provide a robust means of assigning genotypes. In contrast, Sun *et al*, used PCR-RFLP to determine genotypes, which through incomplete digestion of PCR product can lead to misassignment of alleles. This can lead to systematic basis, especially if cases and controls are not genotyped simultaneously. Although we cannot entirely exclude the possibility of such random measurement error as the basis of non-replication of study findings, it seems unlikely to be a major issue.

While there is evidence that difference in genetic frequencies across populations of different racial origin is not generally accompanied by differences in population-specific genetic effects (Ioannidis *et al*, 2004), we cannot exclude this possibility.

On the basis of *in vitro* functional assays, it was asserted that this variant has direct impact of cancer risk via apoptosis. Allele frequencies are, however, very different between populations and the possibility of differential LD in the different populations remains a basis for non-replication. Alternatively, as the study purporting that −652 6N ins/del influences CRC was based on a relatively small sample size to that advocated for GWA analyses, there remains the distinct possibility of a type 1 error coupled with publication bias. If additional studies of these variants are conducted, it opens up the possibility of using meta-analysis to provide the final step in confirming or refuting an association with cancer risk.

## Figures and Tables

**Table 1:** Association between *CASP8* variants D302H (rs1045485) and the ±6N promoter variant rs3834129 with colorectal cancer and results are also shown for the 8q24.21 variant rs6983267

**Gene/locus**	**Variant**	**Genotype**	**Cases (%)**	**Controls (%)**	**OR (95% CI)**
*CASP8*	D302H (rs1045485)	CC	2890 (75%)	2703 (74%)	1.00 (ref)
		CG	894 (23%)	867 (24%)	1.09 (0.75–1.58)
		GG	59 (2%)	61 (2%)	1.13 (0.78–1.63)
		CG/GG	953 (25%)	928 (26%)	0.96 (0.86–1.07)
					*P*_trend_=0.67
					
*CASP8*	−/CTTACT (rs3834129)				*P*_trend_=0.97
		6N ins/ins	995 (26%)	892 (24%)	1.00
		6N del/ins	1897 (49%)	1872 (51%)	0.91 (0.81–1.01)
		6N del/del	987 (25%)	897 (25%)	0.99 (0.87–1.12)
		6Ndel/del +6N del/ins	2884 (74%)	2769 (76%)	0.93 (0.84–1.04)
					*P*_trend_=0.83
					
8q24.21	rs6983267	GG	1102 (31%)	678 (26%)	1.00 (ref)
		GT	1824 (51%)	1297 (50%)	0.86 (0.76–0.97)
		TT	657 (18%)	604 (23%)	0.67 (0.57–0.77)
		GT/TT	2481 (69%)	1901 (73%)	0.80 (0.72–0.90)
					*P*_trend_=1.13 × 10^−6^

Test of HWE: rs3834129, *P*=0.09; rs1045485, *P*=0.37; rs6983267, *P*=0.74. Crude odds ratios are presented. *P*_trend_, test of trend across genotypes. Adjustment for age and sex made no difference to metrics (data not shown).
